# A retrospective cohort study of the application of Santulli enterostomy in neonatal necrotizing enterocolitis

**DOI:** 10.1038/s41598-024-84384-2

**Published:** 2025-01-09

**Authors:** Zhe Fu, Jingmin Zhang, Fanyue Qin, Xinru Wang, Hua Huang, Hongwei Huang, Mingjun Zheng, Peng Wang, Weibo Zhang, Hongguang Shi

**Affiliations:** 1 Department of General Surgery, The Third Affiliated Hospital of Zhengzhou University, Zhengzhou, 450052 China; 2 Department of Pharmacy, The First Affiliated Hospital of Zhengzhou University, Zhengzhou, 450052 China; 3 Henan Province Engineering Research Center of Application & Translation of Precision Clinical Pharmacy, Zhengzhou, 450052 China; 4 Department of Neonatology, The Third Affiliated Hospital of Zhengzhou University, Zhengzhou, 450052 China

**Keywords:** Neonate, Necrotizing enterocolitis, Santulli enterostomy, Nutritional status, Complications, Gastrointestinal diseases, Gastrointestinal diseases

## Abstract

The use of Santulli enterostomy (SE) for necrotizing enterocolitis (NEC) has been limited to a small number of studies involving a small number of patients and no control group. Our study aimed to compare the clinical safety and efficacy of Santulli enterostomy with those of single- or double-lumen enterostomy for neonatal NEC through a retrospective cohort study. One hundred ten patients who met the criteria were divided into an SE group (64 patients) and a conventional enterostomy (CE) group (46 patients). The CE group underwent single- or double-lumen enterostomy. There were no significant differences in complication rates or prognoses between the two groups after either procedure. Although the stoma was positioned higher in the SE group, the length of the unused small intestine (USI) was not significantly different. Multivariate analysis revealed that the length of the USI influenced the likelihood of malnutrition after enterostomy (OR = 1.108, P = 0.008). After stoma closure, compared with those in the CE group, the operation time, intestinal recovery time, fasting time, hospitalization time, intraoperative blood loss volume and the incidence of complications requiring surgical reintervention was significantly lower in the SE group. In conclusion, Santulli enterostomy is not only a safe treatment option for NEC but also an effective method for increasing the length of the small intestine after enterostomy, thereby improving the patient’s postoperative nutritional status. In addition, the procedure is associated with good recovery and a reduced incidence of surgical reintervention after stoma closure.

**Supplementary Information:**

The online version contains supplementary material available at 10.1038/s41598-024-84384-2.

## Introduction

Neonatal necrotizing enterocolitis (NEC) is the most prevalent gastrointestinal emergency in neonates. It can lead to intestinal perforation, multiple organ failure, and even death^1^. NEC occurs in approximately 90% of preterm infants, 50% of whom require surgical intervention^2^. The mortality rate following surgical treatment ranges from 20 to 67%^3^. Routine surgical approaches, such as peritoneal drainage, primary anastomosis, and enterostomy, are commonly used in NEC treatment^4^. The most common enterostomies performed in clinical practice include single-lumen enterostomy, double-lumen enterostomy, Bishop enterostomy, and Santulli enterostomy^5–7^.

Santulli enterostomy involves ostomy of the proximal bowel followed by its end-to-side anastomosis to the distal bowel. It was initially performed in 1961 for the management of congenital intestinal atresia. Subsequently, it was adapted for the treatment of NEC in 1975; a total of five patients were examined, and two survived^8^. In recent years, research on Santulli enterostomy has been expanded to include conditions such as Hirschsprung’s disease and midgut volvulus due to its potential benefits. Despite an increase in the variety of diseases studied, there remains a lack of research on Santulli enterostomy for NEC. Furthermore, relevant studies were limited or lacked control groups^7,9,10^.

In our centre, compared with traditional single- or double-lumen enterostomy, Santulli enterostomy is more clinically beneficial for patients with NEC. Thus, we initiated this research to further evaluate the clinical safety and efficacy of Santulli enterostomy, aiming to provide a reference for surgeons and therefore promote its use for treating NEC.

## Methods

### Inclusion and exclusion criteria

The inclusion criteria were as follows: (1) neonates with NEC presenting clear indications for surgical intervention and (2) neonates who underwent enterostomy.

The exclusion criteria were as follows: (1) neonates who underwent colostomy; (2) neonates who underwent Bishop enterostomy; (3) neonates who underwent small bowel arrangement surgery simultaneously; (4) neonates with lesions affecting the transverse colon or its distal portion, thus the Santulli enterostomy is not possible; (5) neonates with severe intestinal malformations and severe developmental malformations, such as biliary atresia, diaphragmatic hernia, and severe congenital heart disease; (6) neonates who experienced spontaneous intestinal perforation; and (7) neonates who were lost to follow-up after enterostomy or discharged automatically because of family reasons.

### Research participants and surgical procedure

**Fig. 1:**
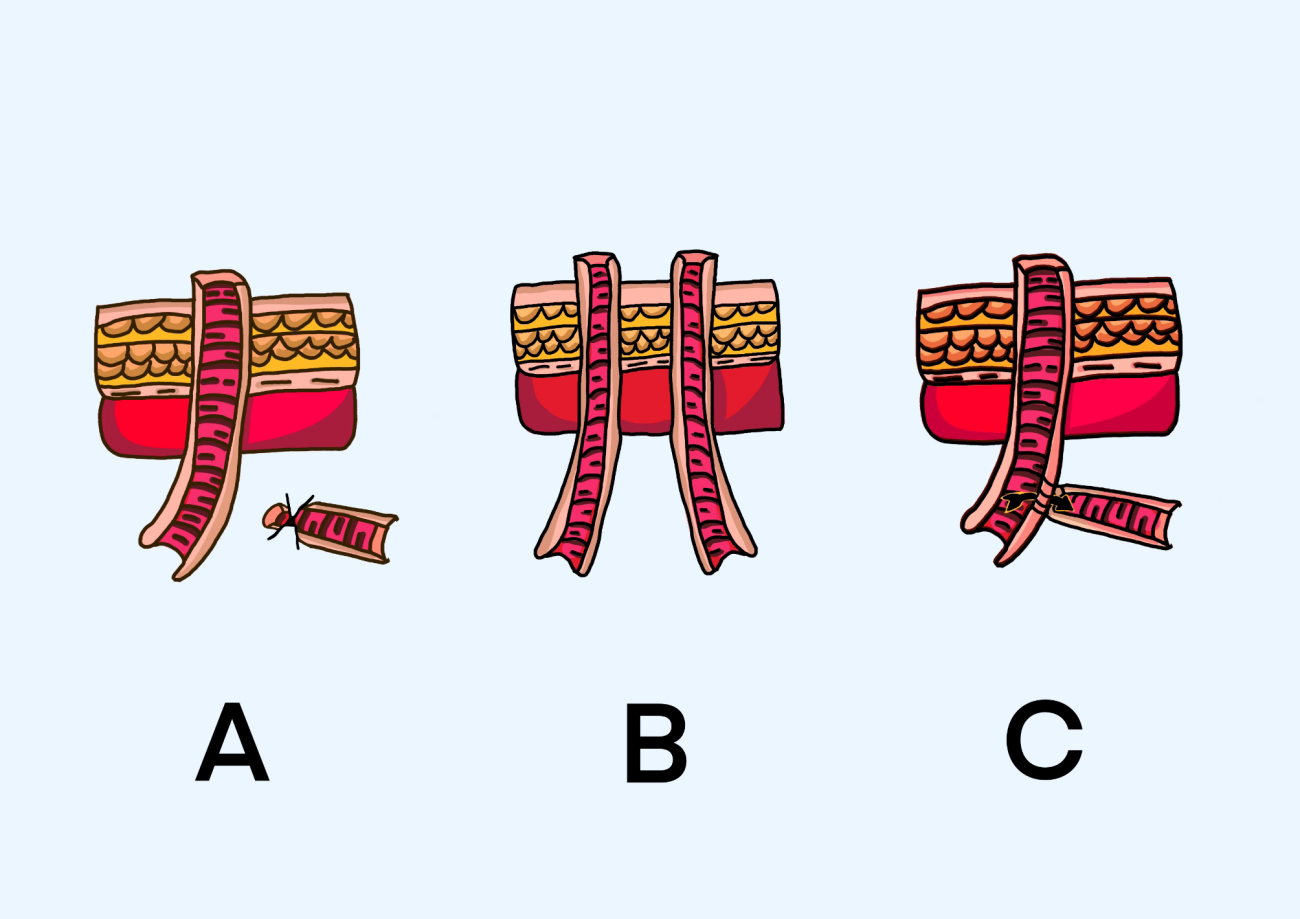
Schematic diagram of the enterostomy methods: (**A**) single-lumen enterostomy; (**B**) double-lumen enterostomy; (**C**) Santulli enterostomy.

The clinical data of 110 neonates with NEC who were admitted to the Third Affiliated Hospital of Zhengzhou University between January 2017 and January 2024 were retrospectively analysed. The neonates were categorized into the Santulli enterostomy (SE) group and the conventional enterostomy (CE) group. The CE group underwent either single- or double-lumen enterostomy. Enterostomy was conducted by a deputy chief physician or higher, and the specific technique was selected on the basis of the patient’s condition as well as the physician’s experience and preference. In the SE group, ostomy of the proximal bowel was performed first, and then its lateral wall was anastomosed to its distal end 5 cm proximal to the ostomy site after the diseased section was resected. While in the CE group, ostomy of the proximal bowel was performed first, and then the distal bowel was closed and either placed in the abdominal cavity or used as the stoma (Fig. 1). This study was approved by the hospital’s Ethics Committee (approval number: 2024-126-01).

### Data collection

The following clinical data of the neonates were collected from the medical records system: (1) demographic information during enterostomy surgery, such as sex, gestational age (GA) at birth, birth weight, age at operation, ventilator use, white blood cell (WBC) count, platelet (PLT) count, C-reactive protein (CRP) level, intraoperative details such as extent of the lesion, status of colon involvement and perforation, and (2) clinical outcomes after enterostomy, such as postoperative hospitalization time, postoperative parenteral nutrition (PN) time and prognoses, including successful stoma closure, death or abandonment and stoma closure after unplanned relaparotomy, postoperative gastrointestinal complications and stoma-related complications.

Additional clinical data were collected from neonates who underwent successful stoma closure surgery. The data are as follows: (1) general information, intraoperative details, stoma height (the length between the stoma and the ileocecal valve), length of the unused small intestine (USI) during enterostomy and the duration between enterostomy and closure (DBEAC). (2) weight-for-age Z score (WAZ) for nutritional status assessment after enterostomy, and clinical outcomes after stoma closure surgery, including operation time, postoperative intestinal recovery time, postoperative fasting time, postoperative hospitalization time, intraoperative blood loss volume, presence of intestinal stenosis during the surgery and surgical reintervention for complications (Clavien-Dindo grade 3 or higher) presenting within one month after surgery according to the Clavien-Dindo classification^11^.

### Related definitions

WAZ = (Measured weight—average weight for the age of the gender)/Standard deviation of weight for the age of the gender. The WAZ score is within the normal range of -2 ~ 2. The unused small intestine (USI) is either the surgically removed part or the distal part of the stoma in the CE group; however, in the SE group, the USI is the surgically removed small intestine because SE maintains intestinal continuity. Extent of necrotic lesions: Focal: the lesion is concentrated in a single intestinal segment; multifocal: the lesion extends to two or more segments of the intestine, but more than 50% of the small intestine is healthy; pan-intestinal: the lesion extensively involves the small intestine and colon, and the remaining healthy intestine accounts for less than 25% of the total length of the intestines^12,13^. In this study, only extensive small intestine involvement, but no more than 50% of the healthy small intestine was also classified as extensive lesions. Short bowel syndrome (SBS) is characterized by the limited absorption capacity of the small intestine due to resection, exclusion, or congenital short bowel, all of which restrict normal growth and development in children and require parenteral nutritional support for more than 42 days^14^. A high-output stoma is a stoma with a defecation volume > 40 ml/(kg·d), accompanied by severe water and electrolyte disturbances requiring correction with intravenous fluid replacement^15^.

### Statistical analysis

SPSS 25.0 statistical software was used for data analysis. Student’s t test or the rank-sum test was used to compare continuous variables between the two groups. The chi-square test was used to compare categorical variables. Univariate analysis and multivariate analysis models were used to analyse the factors influencing nutritional status after enterostomy. A P value < 0.05 indicated statistical significance.

## Results

### Baseline data of patients who underwent enterostomy surgery

On the basis of the predefined inclusion and exclusion criteria, 110 neonates with NEC were enrolled in this study. The patients were categorized into two groups according to the enterostomy method used: the CE group (46 patients) and the SE group (64 patients). There were no significant differences in sex, GA, birth weight, age, ventilator use, WBC count, PLT count, CRP level, or intraoperative details between the two groups (Table 1).

**Table 1:** Baseline data of patients who underwent enterostomy surgery.

Characteristics	CE group	SE group	χ^2^/Z/t	P value
Case number(n)	46	64		
Sex			0.716	0.397
Female	13 (28.3%)	23 (35.9%)		
Male	33 (71.7%)	41 (64.1%)		
GA (weeks)	31.64 (29.46, 35)	31.57 (28.89, 34.29)	-0.497	0.619
Birth weight			5.667	0.129
NWI^a^	8 (17.4%)	4 (6.3%)		
LBWI^b^	14 (30.4%)	31 (48.4%)		
VLBWI^c^	18 (39.1%)	20 (31.2%)		
ELBWI^d^	6 (13.1%)	9 (14.1%)		
Age(days)	13.5 (7.75, 21)	13.5 (7, 21)	-0.227	0.820
Ventilator use			0.007	0.933
Yes	14 (30.4%)	19 (29.7%)		
No	32 (69.6%)	45 (70.3%)		
WBC count (10^9^/L)	7.16 (4.70, 14.75)	6.86 (4.10, 10.25)	-1.067	0.286
PLT count(10^9^/L)	224.35 ± 122.32	226.92 ± 114.45	-0.111	0.912
CRP (mg/L)	46.65 (4.95, 116.25)	48.71 (9.38, 93.95)	-0.015	0.988
Extent of lesion			0.404	0.922
Focal	29 (63%)	39 (60.9%)		
Multifocal	16 (34.8%)	24 (37.5%)		
Pan-intestinal	1 (2.2%)	1 (1.6%)		
Colon involved			0.025	0.875
Yes	18 (39.1%)	26 (40.6%)		
No	28 (60.9%)	38 (59.4%)		
Perforation			3.28	0.070
Yes	19 (41.3%)	16 (25%)		
No	27 (58.7%)	48 (75%)		

Continuous normally distributed data are presented as mean (standard deviations) and nonnormally distributed data are presented as medians (interquartile ranges).

^a^Normal-weight infant: 2500–4000 g; ^b^ low-birth-weight infant: 1500–2500 g; ^c^ very low-birth-weight infant: 1000–1500 g;^d^ extremely low-birth-weight infant: < 1000 g.

### Postoperative outcome data following enterostomy surgery

There were no significant differences in postoperative hospitalization time, postoperative PN time, or rates of gastrointestinal complications and stoma-related complications between the CE and SE groups. A total of ten patients underwent unplanned relaparotomy, including three patients in the CE group and seven patients in the SE group. The prognoses included successful stoma closure, death or abandonment and stoma closure after relaparotomy. There were no significant differences in the prognoses between the two groups (Table 2).

**Table 2:** Postoperative outcomes following enterostomy surgery.

	CE group	SE group	χ^2^/Z	P value
Number of cases	46	64		
Postoperative hospitalization time (days)	23.5 (18.75, 49.25)	28 (16.25, 46.75)	0.968	0.887
Postoperative PN time (days)	19.5 (12.75, 33)	18.5 (13, 32.75)	0.762	0.964
Postoperative gastrointestinal complications	19 (41.3%)	34 (53.1%)	1.498	0.221
Anastomotic leakage	1 (2.2%)	1 (1.6%)	0.000	1
Intestinal obstruction	4 (8.7%)	7 (10.9%)	0.004	0.949
High-output stoma	2 (4.3%)	0	–	0.173
Necrotizing enterocolitis	1 (2.2%)	3 (4.7%)	0.032	0.858
Cholestasis	12 (26.1%)	20 (31.3%)	0.346	0.556
SBS	7 (15.2%)	7 (10.9%)	0.441	0.506
Malnutrition	8 (17.4%)	19 (29.7%)	2.185	0.139
Stoma-related complications	11 (23.9%)	17 (26.6%)	0.099	0.753
Stoma prolapse	4 (8.7%)	4 (6.2%)	0.013	0.908
Stoma retraction	1 (2.2%)	5 (7.8%)	0.738	0.390
Stoma stenosis	2 (4.3%)	1 (1.6%)	0.085	0.771
Poor incision healing	4 (8.7%)	8 (12.5%)	0.399	0.528
Skin damage around stoma	1 (2.2%)	2 (3.1%)	0.000	1
Relaparotomy	3 (6.5%)	7 (10.9%)	0.21	0.647
Prognosis			4.878	0.076
Successful closure of stoma	34 (73.9%)	57 (89.1%)		
Death or abandonment	9 (19.6%)	4 (6.2%)		
Stoma closure after unplanned relaparotomy	3 (6.5%)	3 (4.7%)		

Continuous normally distributed data are presented as means (standard deviations) and nonnormally distributed data are presented as medians (interquartile ranges).

### Baseline data of patients who underwent successful stoma closure surgery

We further compiled the data of 91 neonates whose stomas were successfully closed without relaparotomy and added data on the stoma height and length of the USI during the enterostomy. After analysis, there were no significant differences in the general data of the patients between the two groups. Marked by the ileocecal valve, the median stoma height was 20 cm in the SE group and 10 cm in the CE group, and the difference was significant (P = 0.002), but there was no significant difference in the length of the USI (P = 0.713) (Table 3).

**Table 3:** Baseline data of patients who successfully underwent stoma closure surgery.

Characteristics	CE group	SE group	χ^2^/Z	P value
Number of cases	34	57		
Sex			0.728	0.393
Female	9 (26.5%)	20 (35.1%)		
Male	25 (73.5%)	37 (64.9%)		
GA (weeks)	32 (29.57, 35.82)	31.71 (29.14, 34.36)	-0.677	0.498
Birth weight			6.309	0.093
NWI	7 (20.6%)	3 (5.3%)		
LBWI	12 (35.3%)	31 (54.4%)		
VLBWI	11 (32.4%)	15 (26.3%)		
ELBWI	4 (11.7%)	8 (14%)		
Age(days)	15 (7.75, 22.25)	13 (6.5, 21)	-0.558	0.577
Ventilator use			0.117	0.732
Yes	9 (26.5%)	17 (29.8%)		
No	25 (73.5%)	40 (70.2%)		
WBC count (10^9^/L)	7.02 (4.93, 15.47)	6.88 (4.05, 10.23)	-1.181	0.237
PLT count (10^9^/L)	217.5 (152.75, 316.25)	207 (150, 269)	-0.324	0.746
CRP (mg/L)	61.93 (6.98, 123.08)	47.87 (9.67, 92.91)	-1.153	0.249
Extent of lesion			0.71	0.890
Focal	20 (58.8%)	35 (61.4%)		
Multifocal	14 (41.2%)	21 (36.8%)		
Pan-intestinal	0	1 (1.8%)		
Colon involved or not			0.627	0.428
Yes	16 (47.1%)	22 (38.6%)		
No	18 (52.9%)	35 (61.4%)		
Perforation			3.444	0.063
Yes	14 (41.2%)	13 (22.8%)		
No	20 (58.8%)	44 (77.2%)		
Stoma height (cm)	10 (4.5, 15.5)	20 (11, 37)	-3.055	0.002
Length of USI (cm)	10 (4.5, 15.5)	13 (5, 20)	-0.367	0.713
DBEAC (days)	138 (104.75, 171)	129 (91, 175)	-0.38	0.704

Continuous normally distributed data are presented as means (standard deviations) and nonnormally distributed data are presented as medians (interquartile ranges).

### Nutritional status at stoma closure and postoperative outcomes after stoma closure surgery

There was no significant difference in the WAZ, which represents nutritional status, between the two groups (P = 0.537). The operation time, postoperative intestinal recovery time, postoperative fasting time, postoperative hospitalization time and intraoperative blood loss volume were significantly lower in the SE group than in the CE group. A total of 13 cases of distal intestinal stenosis were identified (7 cases in the CE group and 6 cases in the SE group), with an overall incidence rate of 14.29% (13/91) and no significant difference (P = 0.309). Within one month after stoma closure, only one case of intestinal obstruction was detected in the SE group. However, seven patients in the CE group experienced complications, including two cases of anastomotic leakage, three cases of intestinal obstruction, one case of abdominal haemorrhage and three cases of poor incision healing. The incidence of complications requiring surgical reinterventions was significantly different between the two groups (P = 0.007) (Table 4).

**Table 4:** WAZ at stoma closure and postoperative outcomes following the stoma closure surgery.

	CE group	SE group	χ^2^/Z	P value
WAZ at stoma closure			0.381	0.537
Abnormal	7 (20.6%)	15 (26.3%)		
Normal	27 (79.4%)	42 (73.7%)		
Operation time(min)	99 (69.5, 110)	77 (60, 96.5)	-2.118	0.034
Postoperative intestinal recovery time(days)	2 (1.375, 2.5)	1 (0.5, 1.75)	-3.442	0.001
Postoperative fasting time(days)	5 (4, 7)	4 (2, 5)	-4.024	< 0.001
Postoperative hospitalization time (days)	20 (14, 25.5)	13 (11, 16)	-3.743	< 0.001
Blood loss volume (ml)	5 (4.5,10)	3 (2,5)	-2.98	0.003
Intestinal stenosis			1.035	0.309
Yes	7 (20.6%)	6 (10.5%)		
No	27 (79.4%)	51 (89.5%)		
Surgical reintervention within one month after stoma closure	7 (20.6%)	1 (1.8%)	7.219	0.007
Anastomotic leakage	2 (5.9%)	0	–	0.137
Intestinal obstruction	3 (8.8%)	1 (1.8%)	1.13	0.288
Abdominal haemorrhage	1 (2.9%)	0	–	0.374
Poor incision healing	1 (2.9%)	0	–	0.374

Continuous normally distributed data are presented as means (standard deviations) and nonnormally distributed data are presented as medians (interquartile ranges).

### Univariate analysis and multivariate analyses to identify risk factors for malnutrition at stoma closure surgery

We conducted a single-factor regression analysis of different factors influencing the likelihood of malnutrition at stoma closure, and the factors deemed statistically significant in the univariate analysis were included in the multivariate logistic regression analysis. Multivariate analysis revealed that the length of the USI (OR = 1.108, P = 0.008) was an independent risk factor for malnutrition at stoma closure (Table 5).

**Table 5:** Univariate analysis and multivariate analyses to identify risk factors for malnutrition at stoma closure surgery.

Variables	Univariate analysis			Multivariate analysis		
	OR	95%CI	P values	OR	95%CI	P values
Sex	0.997	0.356–2.793	0.995			
GA	0.931	0.815–1.064	0.296			
Birth weight	1.424	0.811–2.498	0.218			
Age	1.068	1.003–1.136	0.04	1.06	0.984–1.141	0.125
Ventilator use	0.919	0.314–2.686	0.877			
WBC count	0.933	0.837–1.039	0.207			
PLT count	0.977	0.992–1.001	0.15			
CRP	1.003	0.995–1.011	0.467			
Extent of lesion	1.013	0.398–2.578	0.979			
Colon involvement	0.572	0.207–1.578	0.281			
Perforation	0.444	0.135–1.466	0.183			
Stoma height	1.037	1.011–1.064	0.005	0.986	0.935–1.039	0.595
Length of the USI	1.091	1.041–1.142	< 0.001	1.108	1.027–1.195	0.008
Enterostomy method	1.378	0.497–3.817	0.538			

## Discussion

Neonates with NEC often suffer from poor overall health, rapid disease progression, and critical conditions. The timing and method of surgery are closely linked to the prognosis of these patients. Santulli enterostomy involves end-to-side anastomosis of the distal and proximal intestinal tubes at the stoma opening, similar to the combination of primary anastomosis and single-lumen enterostomy. Intestinal inflammation is evident in patients with NEC and often accompanied by intestinal perforation and significant intraperitoneal contamination, both of which can increase the risk of intestinal suture leakage and affect the surgical prognosis^16^. Therefore, primary anastomosis requires careful consideration, and Santulli enterostomy is not recommended for routine use in children with NEC because of the need for anastomosis^17–19^. However, Ming Yue et al*.*^7^ performed Santulli enterostomy in 33 patients diagnosed with NEC, congenital megacolon, intestinal atresia and other diseases, and no anastomotic leakage occurred. In comparison, there were four cases of anastomotic leakage in the control group; therefore, Santulli enterostomy was considered safe and feasible. However, only 26 children with NEC were included in the study, and their intraoperative conditions were not described in detail. In our study, we incorporated intraoperative details such as the extent of the lesion and the presence or absence of colon involvement and perforation as variables into the baseline data to more accurately characterize the fundamental conditions of the two groups. In our study, only two patients experienced intestinal leakage after enterostomy, and the incidence was very low. Meanwhile, there were no significant differences in the prognoses, gastrointestinal complications and stoma-related complications or unplanned relaparotomy rates between the two groups after enterostomy. The comparable incidence of complications and prognoses suggest that Santulli enterostomy is a safe treatment for NEC.

NEC patients with stomas are often at risk of malnutrition. Malnutrition not only affects the prognosis of the disease but also affects the physical and cognitive development of children^20^. Chong et al.^21^ reported that the WAZ at stoma closure was lower than that at the time of enterostomy in 89% of patients, with 42% meeting the criteria for severe malnutrition (WAZ < -3). In the present study, the malnutrition rate was 24.2%, and multivariate analysis revealed that the length of the USI was an independent factor influencing nutritional status. Our study revealed that the median distance between the location of the stoma and the ileocecal region was 20 (11,37) cm in the SE group and 10 (4.5,15.5) cm in the CE group. This difference was statistically significant (P = 0.002), suggesting that the stoma was located higher in the SE group. However, there was no significant difference in the length of the USI between the two groups (P > 0.05). Santulli enterostomy is believed to increase the length of the available small intestine. The Santulli enterostomy operation can maintain intestinal continuity, and the remaining small intestine at the distal end of the stoma can be utilized, especially the ileum, which plays an important role in nutrient absorption^22,23^. Nevertheless, the WAZ did not significantly differ between the groups. The reasons for this result were the limited number of cases studied and the fact that the length of the USI could not be reduced in all children who underwent Santulli enterostomy, particularly those who underwent total resection of the small intestine at the distal end of the stoma. However, given that a longer USI increases the risk of malnutrition, the potential of Santulli enterostomy for reducing malnutrition risk is theoretically supported in cases in which Santulli enterostomy can effectively shorten the USI.

If children with stomas are malnourished, stoma closure surgery is needed to improve their nutritional status, but the optimal timing of stoma closure remains unclear^24–26^. In Santulli enterostomy, plugging the stoma with a sealing device, such as a catheter balloon, can achieve the same effect as stoma closure. There are two opportunities for stoma plugging in cases of Santulli enterostomy. The first opportunity arises when severe malnutrition occurs, allowing observation of nutritional status change after plugging the stoma and it is noted that the stoma should be initially plugged under close medical supervision to avoid harmful consequences. The second opportunity arises when surgeons need to optimize distal bowel function and identify intestinal stenosis prior to stoma closure. Combined with stoma plugging and routine contrast enema, patients with intestinal stenosis after Santulli enterostomy can be well identified, especially those with high output stoma. Intestinal stenosis is a common complication in children with NEC. Regardless of conservative or surgical treatment, intestinal stenosis can develop at different times, mostly within the first three months after NEC. For surgically treated children with NEC, intestinal stenosis mainly occurs in the ascending colon, considering the anatomical location and functional particularity of the ileocecal region^27^. In this study, a total of 13 patients were found to have intestinal stenosis during stoma closure, indicating an incidence of 14.29%, which was consistent with the literature^28^. Six of these cases occurred in the SE group, and the incidence in the two groups was not significantly different.

In the CE group, it is necessary to fully free the proximal and distal intestinal tubes of the stoma during stoma closure surgery and then perform anastomosis, which could theoretically increase the risks of intraperitoneal adherence and anastomotic leakage. In Santulli enterostomy, it is only necessary to identify the presence of distal intestinal stenosis and adhesive obstruction prior to stoma closure. If these conditions are not found, the intestinal tube used for the stoma needs to be dissociated and removed only during the operation, which is simpler. In addition, after single-lumen enterostomy, the distal intestinal tube is disused, and the diameters of the proximal and distal intestinal tubes are significantly different, suggesting a high likelihood of anastomotic stenosis, proximal intestinal tube expansion and torsion^29^. Double-lumen enterostomy can solve the disuse of the distal intestinal tube through chyme reinfusion, but the stoma nursing operation is complicated. Santulli enterostomy maintains the continuity of the intestine, promotes the circulation of the intestinal contents to the distal end and restores the function of the distal intestinal tube of the stoma. Therefore, the operation time and the blood loss volume of stoma closure surgery were lower in the Santulli enterostomy group compared with those in the CE group in the study. The results also showed that patients recovered more rapidly in the SE group after stoma closure.

Our study was retrospective in nature and may introduce bias into the research data. Furthermore, there is currently no objective index reflecting the degree of intestinal inflammation to guide surgeons in selecting Santulli enterostomy, particularly when the risk of intestinal leakage is considered. Therefore, doctors may rely on their experience when making such decisions.

## Conclusion

Santulli enterostomy is not only a safe treatment option for NEC, even in cases involving intestinal tube necrosis or perforation, but also an effective method for increasing the length of the small intestine in most cases, thereby improving the postoperative nutritional status in children. Additionally, it has the potential to yield greater benefits in terms of stoma closure, particularly by reducing the likelihood of surgical reinterventions.

## Electronic supplementary material

Below is the link to the electronic supplementary material.

Supplementary Material 1

Supplementary Material 2

## Data Availability

Data is provided within the manuscript or supplementary information files. If someone wants to request the data from this study could contact the corresponding author.
