# Supplementary material for: A retrospective cohort study of the application of Santulli enterostomy in neonatal necrotizing enterocolitis
Source: Sci Rep. 2025 Jan 9;15:1475. doi: 10.1038/s41598-024-84384-2 (PMC11718129; doi:10.1038/s41598-024-84384-2)
Supplement: Supplementary file 2 — Supplementary Material 2 [file 41598_2024_84384_MOESM2_ESM.docx]

**Consent statement**

Due to the retrospective nature of the study, the Ethics Committee of Third Affiliated Hospital of Zhengzhou University waived the need of obtaining informed consent.
